# Whole Body Vibration-Induced Omental Macrophage Polarization and Fecal Microbiome Modification in a Murine Model

**DOI:** 10.3390/ijms20133125

**Published:** 2019-06-26

**Authors:** Jack C. Yu, Vanessa L. Hale, Hesam Khodadadi, Babak Baban

**Affiliations:** 1 Children’s Hospital of Georgia, Medical College of Georgia, Augusta University, Augusta, GA 30912, USA; 2 Department of Oral Biology, College of Dental Medicine, Augusta University, Augusta, GA 30912, USA; 3 Veterinary Preventive Medicine, Ohio State University, College of Veterinary Medicine, Columbus, OH 43210, USA

**Keywords:** WBV, whole body vibration, obesity, type II diabetes, macrophage, microbiome, innate immunity

## Abstract

Human nutrient metabolism, developed millions of years ago, is anachronistic. Adaptive features that offered survival advantages are now great liabilities. The current dietary pattern, coupled with massively reduced physical activities, causes an epidemic of obesity and chronic metabolic diseases, such as type 2 diabetes mellitus. Chronic inflammation is a major contributing factor to the initiation and progression of most metabolic and cardiovascular diseases. Among all components of an innate immune system, due to their dual roles as phagocytic as well as antigen-presenting cells, macrophages play an important role in the regulation of inflammatory responses, affecting the body’s microenvironment and homeostasis. Earlier studies have established the beneficial, anti-inflammatory effects of whole body vibration (WBV) as a partial exercise mimetic, including reversing the effects of glucose intolerance and hepatic steatosis. Here for the first time, we describe potential mechanisms by which WBV may improve metabolic status and ameliorate the adverse consequences through macrophage polarization and altering the fecal microbiome.

## 1. Introduction 

Whole body vibration (WBV) is an exercise mimetic; it decreases the inflammatory response and can reverse many symptoms of type II diabetes mellitus (T2DM), such as polyuria and polydipsia. It also significantly improves glucose metabolism measured by a glucose tolerance test and Hb A1C [1]. Recent reports also reveal marked improvement in hepatic lipid content, decreasing it three-fold [2]. However, how WBV achieves these beneficial changes remains unclear. Glucose transport in myeloid cells mediated by IL-3 appeared as early as the mid-1990s [3]. Clinical investigations have also confirmed that perturbation in glucose metabolism is present in acute myeloid leukemia [4,5]. Importantly, it has become clear that a complex regulatory network exists, linking glucose metabolism to both myeloid and lymphoid homeostasis. Increased glucose availability to macrophages might initiate a feed forward loop that fosters inflammation and exacerbates insulin resistance and hyperglycemia [6]. Within this complex network, macrophages display great functional plasticity and the M1/M2 nomenclature now classifies macrophages into cells with pro-inflammatory (M1) or anti-inflammatory (M2) properties [7]. In mammals, this polarization and other innate immune functions link closely to the largest microbial load: The microbiota of the alimentary canal, with an intimate and reciprocal relationship, in that microbiota affects innate immunity and vice versa [8]. The impact and importance of this relationship between microbiota and innate immunity is indisputable [9,10]. Therefore, there are two objectives to this study. The first is to investigate the changes in macrophage type in blood and adipose tissues by characterizing the macrophage profiles in these tissues before and after WBV, using a well-established murine T2DM model. The second objective, as an initial step towards a proximate mechanistic exploration in trying to establish casual links between WBV and downstream effects, we seek to document fecal microbiome changes related to WBV.

The three hypotheses tested are as follows:

1. M1 predominates in the abdominal and blood macrophages in T2DM mice;
2. WBV can cause macrophage polarization from M1 towards M2, decreasing pro-inflammatory cytokines and increasing anti-inflammatory cytokines;
3. WBV causes alterations in both the alpha and beta diversity of fecal microbiome.

As the prevalence and incidence of T2DM and obesity continue to increase in the US population, effective adjuncts to standard therapy such as WBV are gaining momentum. It is critically important that we explore the mechanism underpinning this promising potential therapeutic modality. 

## 2. Results

### 2.1. Macrophage Polarization 

**Figure 1:**
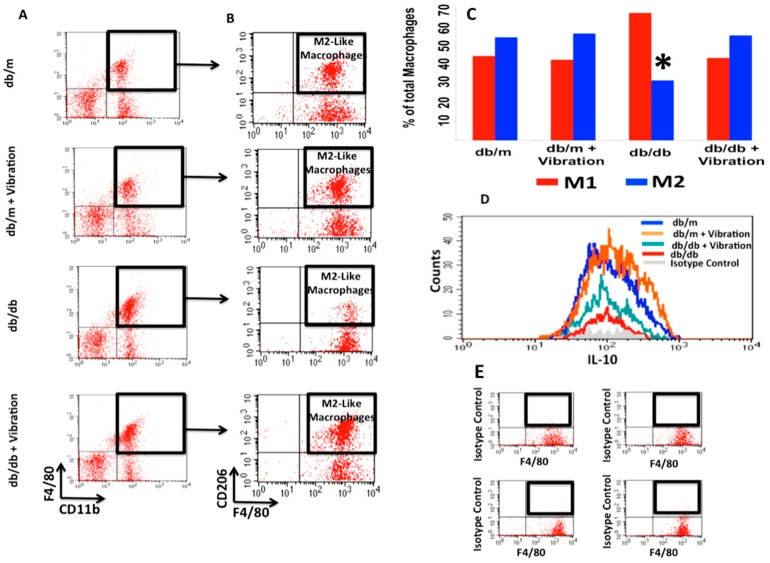
Whole Body Vibration (WBV) skews macrophage polarization from pro-inflammatory (M1) to anti-inflammatory (M2) in adipose tissue. Panels (**A**) and (**B**) show the flow cytometry analysis of adipocytic macrophages, demonstrating an increase in the frequencies of M2 macrophages after WBV. Panel (**C**) illustrates the ratio of M1/M2 adipocytic macrophages in db/m mice versus db/db either subjected to WBV or no intervention (*p < 003). Panel (**D**) displays the profile of IL-10 expression in macrophages from omental fat in db/db and db/m mice with and without WBV. The db/db omental macrophages showed a lower (25%) baseline IL-10 level than db/m. With WBV, IL-10 level increased 2× in db/db but not the db/m omental macrophages. Panel (**E**) exhibits the isotype control for technical and specificity of antibodies.

As shown in Figure 1, analysis of macrophages in all test groups revealed a significant increase in M2 macrophage count (functional M2 macrophages: CD11b+, F4/80+, CD206+, IL-10+) in db/db mice subjected to WBV. The anti-inflammatory IL-10 level increased in the control db/m group as well. The resting IL-10 level was higher in db/m mice, consistent with a higher proportion of M2 when compared with db/db. WBV restored the M2 level in db/db mice to the resting level of M2 in db/m, but not as high as the db/m with WBV. These findings are consistent with our hypothesis that WBV induces macrophage polarization to M2 type in a diabetic mouse model. It also revealed that WBV elevated IL-10 levels in normal controls, as well as db/m.

### 2.2. WBV Induced Changes in db/db Microbiome

**Figure 2:**
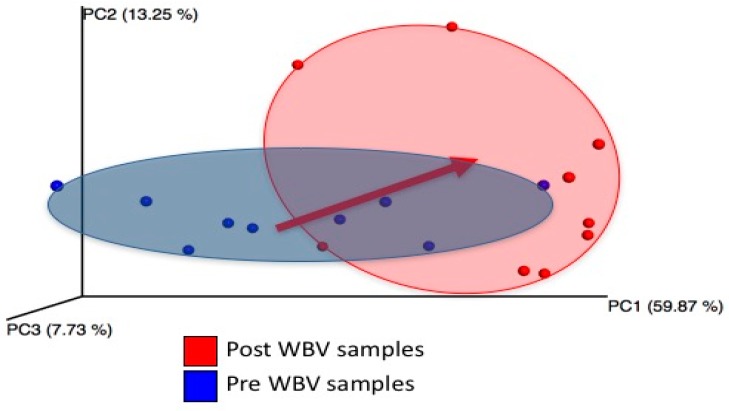
Changes in beta diversity (weighted UniFrac), due to WBV. Each point represents microbial community composition (weighted by species abundance) within an individual mouse. Distance between points indicates how similar microbial communities are between hosts and time points. Microbial composition shifts post WBV.

**Figure 3:**
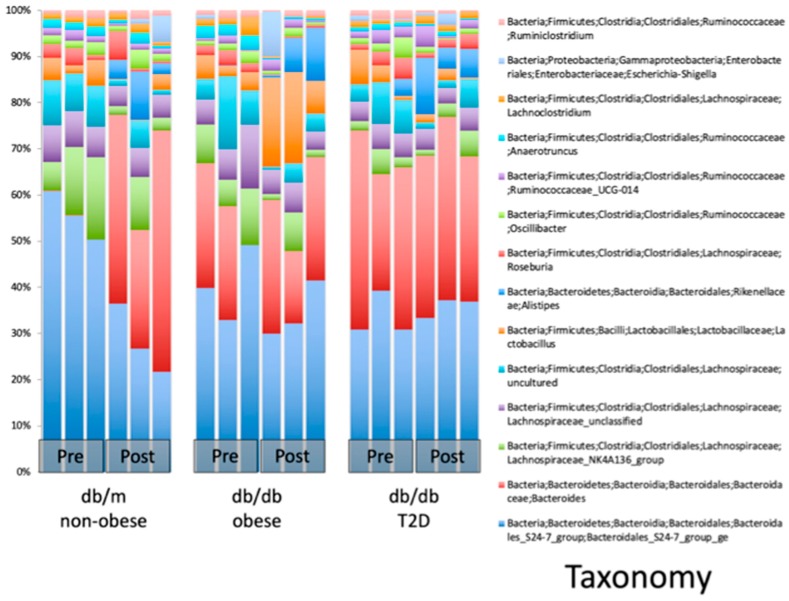
Taxonomic classification of the most abundant species in each mouse. Each column represents the microbial community within a single mouse. Post WBV, db/m mice showed an increase in microbes in the genera Bacteroides and Alistipes. The db/db obese mice showed an increase in microbes in the genera Lactobacillus and Alistipes. The db/db type 2 diabetes mellitus (T2DM) mice also showed an increase of microbes in the genus Alistipes.

**Figure 4:**
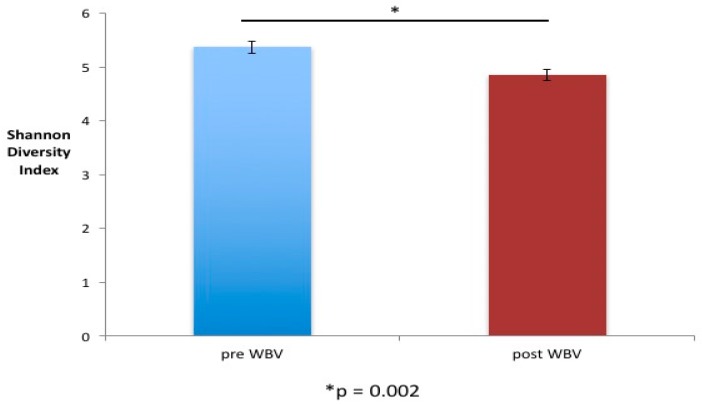
Alpha diversity changed with WBV. There was a significant decrease in gut microbial diversity (Shannon Diversity Index) between pre- and post-WBV samples (Error bars = standard error). * Statistically significant difference between two groups.

We observed significant changes in the microbial composition and diversity of eight-week-old male db/db mice after six weeks of WBV (20 min per day, five days a week) (Figure 2, Figure 3 and Figure 4). Microbial composition shifted post WBV (Figure 2), and distances between pre- and post-WBV samples were significantly farther than distances within pre- or post-samples (*t*-test; Bonferroni-corrected *p*-values < 0.005) Microbes in the genera Alistipes (Family: Rickenellaceae), which are anaerobic, pigment forming, Gram-negative rods, significantly increased (*p* = 0.02, 17-fold increase) post WBV (Table 1 and Figure 3). Alpha diversity (Shannon diversity index) was significantly reduced post WBV (*p* = 0.002) (Figure 4). Higher gut microbial diversity has been commonly associated with health, and exercise can induce increases in microbial diversity. However, the reduced diversity observed in this study post WBV may result from an increase in the beneficial species that produce short-chain fatty acids rather than ethanol (Figure 3 and Figure 4).

## 3. Discussion 

The prospect of WBV as an adjunct to improve musculoskeletal health and its potential benefits on several metabolic diseases, such as diabetes and hypertension, have recently attracted substantial interest [11]. Despite a certain level of controversy, all studies generally support the notion that WBV may improve the inflammatory indices and help to re-establish the immune balance and homeostasis [12]. In fact, our recent findings showed that WBV may be capable of de-escalating inflammation by reducing IL-17^+^ helper T cells and elevating Fox P3^+^ regulatory T cells [1]. While the biochemical and physiological improvements from WBV as an exercise mimetic are indisputable, how WBV achieves such effects is likely multi-faceted and largely unknown. Furthermore, our recent data have pointed to potential alterations in the chemical composition of the portal blood after WBV, evidenced by the beneficial effects seen in the liver [2]. The current experiments seek to answer if WBV can alter fecal microbiome and whether it causes polarization of omental macrophages from M1 to M2. 

Macrophages are essential components of the innate immune system, playing a crucial role in the activation and regulation of immune responses. Macrophages apply their regulatory effects through three major mechanisms, including phagocytosis, antigen presentation, and cytokine production [13]. Among all these three macrophage functions, the cytokine signaling is directly downstream of macrophage polarization. Macrophages are classified based on their phenotype and function in pro-inflammatory M1 and counter-inflammatory M2 types. Several factors including host microenvironment and metabolic interaction can affect the polarization process of macrophages [14]. 

Our data showed that WBV not only could skew the macrophage polarization towards M2, the counter-inflammatory macrophages, it also altered the microbiome in the digestive tract. In fact, this is the first study to report a documented potential cross talk between microbiome and innate immunity through macrophage polarization mediated by WBV. The remodeling of microbiome was in both the alpha and beta diversity of the gut microbiome following WBV, with a massive increase in alistipes. *Alistipes* belong to the Rikenellaceae family of class Bacteroidia and are present in very small qualities in the typical intestinal microbiome of C57/B6 mice after weaning [15]. They are non-alcoholic fermenters and produce short-chain fatty acids (SCFA), such as acetoacetate and butyrate, known to be fuel for the gut flora, highly anti-inflammatory, and capable of reversing adverse effects of a high-fat diet [16]. Of great interest is the recent observation that alistipes increased when mice were fed beta-glucans, which induces higher levels of SCFA [17]. Their presence has also been detected in hibernating mammals that undergo extensive nutritional and microbiological adaptations during the winter months [18]. It is very likely that the gut microbiome change is the proximate cause of improved hepatic steatosis in T2DM mice after WBV. This is in agreement with the anatomy and clinical observations [19]. However further investigations are required to confirm this in the case of WBV.

The current studies tested if WBV could alter the polarization of omental macrophages. Macrophages are the principle component of innate immunity through their resident antigen-presenting function. The sequencing of the WBV effects is still unclear: Is M2 polarization antecedent to intestinal microbiome changes or vice versa? In this complex network of regulatory and counter-regulatory nodes, there is much still to be worked out at multiple levels from changes in gene expression to cell, tissue, and organ level remodeling.

In summary, it is noteworthy to emphasize that inflammatory responses resulting from a variety of diseases, including diabetes and obesity, are integral to the pathogenesis of these diseases. There is a self-perpetuating, vicious cycle: Metabolic dysregulation and tissue damage cause inflammation, and inflammation causes more tissue destruction and metabolic dysregulation. Sustained inflammation underpins a wide range of diseases from cardiovascular and metabolic dysfunction (e.g., cardiorenal diseases, diabetes), cognitive impairment (e.g., dementia) to several levels of neoplastic-dysplastic transformations (e.g., cancer). These current findings support the notion that WBV has the potential to alter the microbiota in a way that triggers innate and mucosal immunity to produce anti-inflammatory responses, down-regulating the hyper-inflammatory state and reversing the adverse consequences. More studies are required to solidify this novel approach, which can be a very affordable and an effective therapeutic modality in the prevention and treatment of many diseases, including diabetes and obesity.

## 4. Materials and Methods 

All procedures followed the Public Health Service Guide for the Care and Use of Laboratory Animals and Augusta University guidelines. 

### 4.1. Macrophage Polarization Experiments

Two groups of male mice were used (*n* = 9): Homozygote db/db mice (*n* = 6) and heterozygote db/m as controls (*n* = 3). The db/db mouse model of leptin deficiency is currently the most widely used mouse model of type 2 diabetes mellitus (T2DM). This mouse has a mutation in the gene encoding the leptin receptor, and leptin deficiency entails susceptibility to obesity, insulin resistance, and T2DM. The db/m is the group of non-diabetic heterozygous control mice [20]. The animals were acclimated and given a regular diet and water. The diet was the standard natural-ingredient diet for rodents and was purchased from Teklad (Envigo, Madison, WI, USA). The diet fulfills many everyday needs of laboratory animal colonies for breeding, growth, and maintenance. These diets consist of relatively unrefined agricultural commodities, such as grains, grain by-products, concentrated plant protein sources, animal proteins, and vitamins, minerals, and fats. Animals were subjected to WBV based on the experimental design with a frequency of 30 Hz; amplitude: 3 mm, 0.5G to 1.5G for 20 min/day for 7 days per week for 4 weeks [1,2]. The amplitude is the maximum displacement distance of the platform surface on which the mice were placed. The frequency of 30 Hz and magnitude of 0.5–1.5× gravitation (4.9 to 14.7 m/sec^2^) were verified by an accelerometer. The animals were sacrificed at the end of 4 weeks. Abdominal adipose tissues were collected. The adipose tissue samples were first cut into small pieces, digested with collagenase, and filtered through a 100 μm cell strainer to make a single cell suspension.

### 4.2. Analytic Flow Cytometry

For flow cytometry analysis, all single cell suspensions were stained with macrophage markers, CD68, CD206, and TNFa cells to quantify M1 and CD68, CD206, and IL-10+ for M2. Briefly, cells from abdominal adipose tissue samples were stained with CD11b and an acceptable macrophage marker F4/80 to select macrophages. CD11b+ and F4/80+ cells were further incubated with an antibody against CD206, a c-type lectin with a high affinity for mannose, to select M2 macrophages. To confirm their functionality, CD11b+, F4/80+, and CD206+ were further stained intracellularly with IL-10 to indicate the M2 phenotype. A four-color flow cytometer (FACSCalibur, BD Biosciences, San Jose, CA, USA) and CellQuest software (BD Biosciences) were used for analysis as we described previously [21]. Isotype-matched controls were analyzed to set the appropriate gates for each sample. For each marker, samples were analyzed in duplicate. To minimize false-positive events, the number of double-positive events detected with the isotype controls was subtracted from the number of double-positive cells stained with corresponding antibodies (not isotype control), respectively. Viable cells were visibly differentiated from debris by gating on live cells with high forward scatter (FSC) and positivity for specific antibodies. Single stains were performed for compensation controls, controls to check for fluorescence spread, and isotype controls were used to determine the level of nonspecific binding. Cells expressing a specific marker were reported as a percentage of the number of gated events.

### 4.3. Fecal Microbiome Changes

Eight week-old male db/db mice (*n* = 9) were purchased from a commercial vender (Charles River, Wilmington, MA, USA) and fed a regular diet ad libitum. All mice were obtained from the same vender and housed in three cages with no changes in diet. After acclimatization, they went through daily WBV at 30 Hz, for 20 min, 5 days per week for 6 weeks. The amplitude was from 0.5 to 1.5 g. The stool samples were collected before and after the entire WBV course. 

For fecal sample collection, we followed the standard protocol: 1–2 pellets per mouse were collected fresh, immediately frozen, and stored in a –80 °C freezer until analysis. 

### 4.4. DNA Extraction, Library Preparation, Sequencing

DNA extraction and library preparation were performed by the Mayo Clinic Microbiome Lab (Rochester, MN, USA). The Mo Bio PowerSoil DNA isolation kit (Mo Bio Laboratories, A Qiagen Company, Carlsbad, CA, USA) was used for DNA extraction. The V3–V5 region (forward primer 357F, reverse primer 926R) of the 16S rRNA gene was sequenced on an Illumina MiSeq at the Mayo Clinic Medical Genomics Facility, using a MiSeq (2 × 300, 600 cycles, Illumina Inc., San Diego, CA, USA). After quality-filtering the sequencing files, high resolution assignment of operational taxonomic units (OTUs) was done using a sub-operational taxonomic unit (sOTU) approach, Deblur, to identify microbial sequences [22]. Mothur was then used for taxonomy calling with the silva128 database [23], assigning microbial sequences at the genus level. The TRE file was constructed using FastTree [24].

### 4.5. Statistics

Flow cytometric data were examined using the analysis of variance, followed by a Newman–Keuls post hoc test to establish significance (*p* < 0.05) among groups. Data are reported as means ± SEM. Alpha diversity (Shannon diversity index) and beta diversity metrics (Weighted UniFrac) were generated in QIIME 1.9.1. Alpha diversity was compared using an ANOVA in RStudio (Version 1.1.456). Differential taxonomic abundance was assessed using the group_significance.py command in QIIME 1.9.1. [25].

## 5. Conclusions

The four key findings of the above series of experiments are:

1. The baseline ratio of omental M1 to M2 macrophages in T2DM mice is 2:1;
2. WBV can cause M1 to M2 polarization in both control and T2DM mice;
3. WBV restores M2 levels in T2DM to near baseline levels of the normal control;
4. WBV alters the fecal microbiome in T2DM mice, increasing bacteroides, especially those belonging to the genus *Alistipes* of the Rikenellaceae family, which increased by 17.75 times.

## Figures and Tables

**Table 1:** Taxa with the most significant increases post WBV. Alistipes increased by 17 times. This analysis combined the pre-/post-WBV results from mice in all cages.

Pre WBV Mean Reads	Post WBV Mean Reads	FDR *p* Value	Taxonomy
703.22	12479.56	0.02	Bacteria; Bacteroidetes; Bacteroidia; Rikenellaceae; Alistipes
943.33	3724.56	0.03	Bacteria; Bacteroidetes; Bacteroidia; Bacteroidales; Bacteroidales_S24-7_group; Bacteroidales_S24-7_group_ge
2746.78	11404.44	0.03	Bacteria; Bacteroidetes; Bacteroidia; Bacteroidales; Bacteroidales_S24-7_group; Bacteroidales_S24-7_group_ge
